# The Research of Feasibility and Efficacy of Radiofrequency Ablation in Treating Uterine Fibroids

**DOI:** 10.1097/MD.0000000000001956

**Published:** 2015-10-30

**Authors:** Xin Luo, Shan-rong Shu, Xue-feng Ma, Han-lin Shuai

**Affiliations:** From Department of Obstetrics and Gynecology, The First Affiliated Hospital of JiNan University, Guangzhou, People's Republic of China.

## Abstract

To explore the feasibility and efficacy of radiofrequency ablation in treating uterine fibroids.

Ninety patients with multiple uterine fibroids, who had undergone hysterectomy were included in the study. After the uterus was resected, the temperature of 60, 80, 100°C were adopted to ablate the in vitro fibroid with each temperature dealing with 30 patients. Simultaneously, 5 patients were included, whose in vivo fibroid were ablated with the temperature of 100°C before the fibroids were removed after laparotomy. After the fibroids were ablated, the smooth muscle in the ablated center (group A), the ablated edge (group B) and 1 cm away from the ablated edge (group C) were taken. Then, the samples were stained with hematoxylin and eosin (HE) to examine the histopathological changes, and immunohistochemistry was performed to detect the expression of estrogen receptor (ER) and progesterone receptor (PR).

After radiofrequency ablation, the ablated lesions were round, toast tan, and dry on gross appearance. There were no obvious tissue carbonization and there were distinct boundary from periphery tissue. In vitro: On automated analysis, the average optical density of ER and PR in group A, B, and C was lower than the control group (*P* < 0.05), and which were gradually raised with the increased distance to electrode. In the same treatment group, ER optical density was gradually decreased with the increased temperature among 3 different groups. The PR optical density was decreased with the increased temperature under different temperatures in group A and group B, there was significant difference among groups (*P* < 0.05). But in group C, there was no difference in PR expression among the temperature of 60, 80, and 100°C (*P* > 0.05). In vivo: Compared with the control group, the average optical density of ER and PR were significantly different among group A, B, and C (*P* < 0.05), what's more, it was gradually raised with the increased distance to electrode.

After radiofrequency ablation, the tissues displayed coagulative necrosis, and decreased ER and PR expression. Radiofrequency ablation may be considered a minimally invasive alternative for those women who wish to retain their reproductive potential. Eighty degree Celsius was expected to be the optimum temperature in radiofrequency ablation treatment of uterine fibroid.

## INTRODUCTION

Uterine fibroid (leiomyoma or myoma) is one of the most common benign tumors in women. They are mostly asymptomatic, but frequently cause menstrual abnormalities, pelvic mass, infertility, and pressure symptoms, leading to an impaired quality of life.^1^ Uterine fibroids are present in as many as 25% to 30% of women in the general population,^2^ and are symptomatic in about 70% to 80% of these women among the age group 30% to 50 years, with the rate of hysterectomy being 60%.^3^ While asymptomatic uterine fibroids can be safely observed, myomectomy has been the standard treatment in symptomatic patients. Hysterectomy may be performed via abdominal route, laparoscopically, or hysteroscopically.^4^ Hysterectomy is an option for those who have completed their family, while myomectomy is the time-tested approach in women wishing to retain reproductive function. In order to circumvent the complications associated with traditional surgery, several minimally invasive, or nonsurgical methods are currently being researched. Uterine artery embolization and magnetic resonance guided focused ultrasound are in common use.

Radiofrequency ablation utilizes high temperature with 85 to 100°C generated by high frequency alternating current cause tissue coagulative necrosis in situ.^5^ It has the advantages of higher efficacy, minimal trauma, and easy accessibility.^6,7^ Radiofrequency ablation has been successful in the treatment of neoplasms of the liver, lung, breast, adrenal gland, prostate, and central nervous system.^8–12^ It's role on uterine fibroid and related mechanism were rarely reported. In our study, we intended to elucidate the pathological changes and estrogen receptor (ER)/progesterone receptor (PR) expression of fibroids treated with in vitro and in vivo radiofrequency ablation. We wanted to establish the safety and objective evidence of efficacy of radiofrequency ablation (RFA) by histopathological examination.

## DATA AND METHODS

### Clinical Data

This observational study was approved by the Institutional Ethics Committee, and informed consent was taken from all subjects. For the in vitro experiments, we chose 90 subjects with multiple uterine fibroids and admitted to the gynecology department of the first affiliated hospital of JiNan University. The mean (standard deviation [SD]) age was 46.5 (5.8) years, the mean weight (SD) was 54 (4) kg, and all of them had standard indications for trans-abdominal hysterectomy. After hysterectomy, the fibroids were ablated in vitro at a temperature of 60, 80, and 100°C from uterine of 30 subjects at each temperature. For the in vivo experiments, we chose 5 patients with uterine multiple fibroids who were admitted in the same department. The mean (SD) age was 46.5 (5.6) years and the mean (SD) weight was 53 (3.5) kg. With the patients’ consent, we ablated the fibroids in vivo at a temperature of 100°C before the fibroids were resected during laparotomy. None of the patients had any history of any serious health condition, including any pelvic disease. Additionally, none of them had undergone any treatment for their fibroids in the previous 3 months. All the uterine were resected in the first half of the menstrual cycle. The diagnosis of uterine fibroids was confirmed in all the specimens by a postoperative pathological examination. There was no fibroid degeneration, and the endometrium appeared to be in the proliferative phase, in all specimens.

### Methods

#### Radiofrequency Ablation Apparatus

**FIGURE 1:**
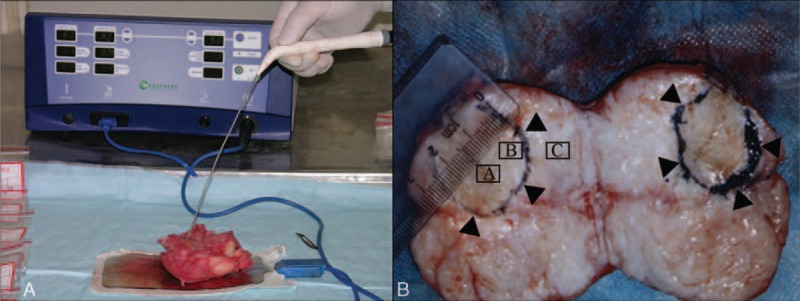
MSI S-500 radiofrequency ablation apparatus and the procedures using which the fibroids were ablated. (A) MSI S-500 radiofrequency ablation apparatus was used to ablate the resected uterus. (B) The circle showed the scope of the ablated lesion, the diameter of the ablated lesion was 2.5 cm. “A” represents the center of the ablated lesion, “B” represents the edge of the ablated lesion, and “C” represented the area 1 cm away from the edge of the ablated lesion.

We used MSI S-500 radiofrequency ablation apparatus, manufactured by MaiDe Medical Science and Technology Ltd (China). The work frequency was (550 ± 50) Hz, and the output power of generator was 0 to 60 W. While treating uterine fibroids, an output power of 0 to 30 W was used for a time limit of 5 minutes. In the in vitro experiments, 3 temperatures were studied (60, 80, and 100°C). In the in vivo experiments, the ablation was performed at a temperature of 100°C. The ablation was performed using a sharp knife/needle tool as a treatment electrode of radiofrequency ablation apparatus. A section of 0.5 to 1.0 cm was exposed, and rest of the area was packed with insulation material (Figure 1A).

#### Treatment Methods

##### In Vitro Experiments

Ninety patients underwent hysterectomy under combined spinal–epidural anesthesia. After the uterus was resected, the subserous or intramural fibroids protruding from the serosal surface with a diameter of 3 to 5 cm were ablated. The needle was inserted into the center of the fibroid, and radiofrequency ablation was done for a duration of 5 minutes.

##### In Vivo Experiments

In 5 patients, after combined spinal–epidural anesthesia, the uterus was exposed by laparotomy. Before hysterectomy, the in vivo fibroids with a diameter of 3 to 5 cm was ablated at a temperature of 100°C. The needle was inserted into the center of fibroid, and the radiofrequency ablation procedure was performed for a maximum duration of 5 minutes and maximum output power of 30 W. After ablation, the uterus was resected.

#### Preparation of Specimen

The ablated fibroid was cut along the needle tract. Sections were taken from the center of the ablated fibroid (group A), the edge (group B), and 1 cm away from the edge (group C; Figure 1B). Sections from the nonablated fibroids in the same uterus constituted the control group. Tissue sections measuring 1.0 × 1.0 × 0.3 cm^3^ were fixed with formalin, dehydrated, and embedded in paraffin, after which 3 thin sections of 4 to 5 μm thickness were made for microscopic examination using hematoxylin and eosin (HE) staining. Immunohistochemical analysis was performed to detect the ER and PR expression.

#### Observation Item and Result Determination

We observed the histomorphological changes of the 3 treatment groups under optical microscope and examined the ER and PR expression using immunohistochemical staining. Brown staining of the nucleus was considered to be positive for ER and PR. For each case, the entire stained section was scanned and 10 visual fields were randomly chosen. We used the high resolution color automatic pathological graphic analysis system to detect the optical density of positive cells.

#### Statistical Analysis

All statistical analyses were performed by using SPSS13.0 (Version 13.0; SPSS, Inc., Chicago, IL). Comparisons among all groups were performed with the 1-way analysis of variance (ANOVA) test. If statistical significance was found, the Tukey post hoc test was used. A value of *P* < 0.05 was considered statistically significant.

## RESULTS

### Tissue Morphological Changes of Uterine Fibroids Treated in Vitro With Radiofrequency Ablation

**FIGURE 2:**
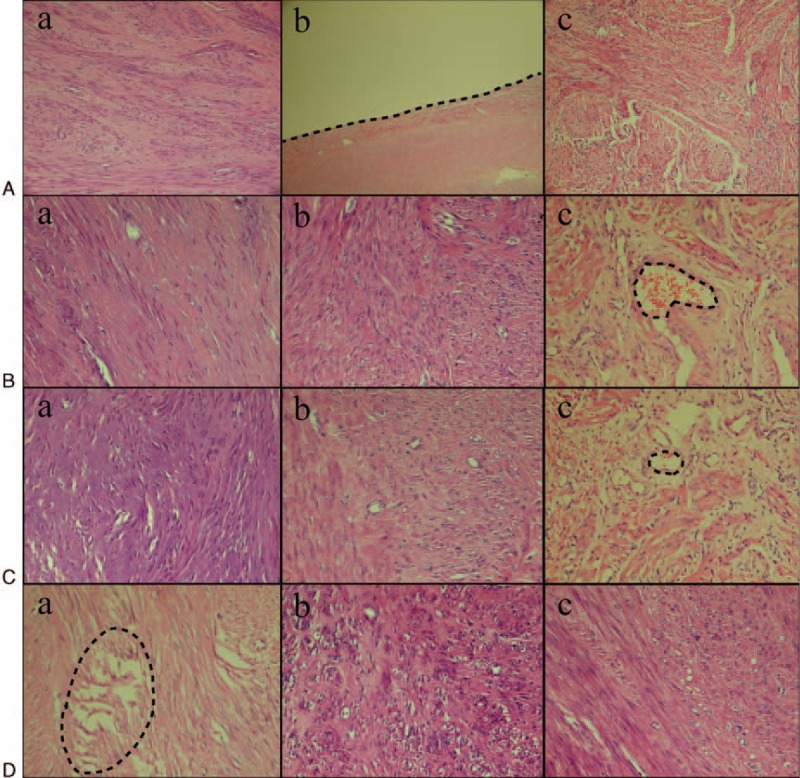
Histological changes in the fibroid treated at different temperatures (×200). (A) Histological change of fibroid ablated in vitro at a temperature of 60°C. (a) Focal coagulation necrosis, with part of the muscle fibers being visible. (b) Bleeding line around the edge of the ablated lesion (the black line). (c) No degeneration, necrosis, or inflammatory cell infiltration in the fibroid tissue. (B) Histological changes in the fibroid ablated in vivo at a temperature of 80°C. (a) Coagulation necrosis, damaged muscle fiber structure, karyoclysis, and reduced nuclear color. (b) Lesser tissue necrosis compared with group A, damaged muscle fiber structure, but many cell nuclei visible. (c) No muscle cell necrosis and inflammatory cells filtration. There was a patent blood vessel in the center of lesion, with presence of red blood cells (the black circle). (C) Histological change in fibroid when ablated in vitro at a temperature of 100°C. (a) Coagulative necrosis, damaged muscle fiber structure, and karyorrhexis. (b) Partial regional necrosis, which was lesser compared with the group A. (c) No obvious fibroid cells degeneration, necrosis, but several small patent blood vessels with red blood cells (the black circle). (D) Histological change in fibroid when ablated in vivo at a temperature of 100°C. (a) Coagulative necrosis, electric shock like fracture, damaged muscle cell structure, and karyorrhexis (the black circle). (b) Focal coagulative necrosis, the extent of which was lesser compared with group A. (c) No obvious degeneration, necrosis, and inflammatory cells infiltration in the leiomyoma cells.

After ablation at all 3 temperatures, the uterine fibroids were round, dry, and toast tan in color, with no obvious tissue carbonization. There was a clear boundary of periphery tissue. The HE stained tissue was observed under optical microscope. In the group of samples ablated at 60°C (Figure 2A), group A showed focal coagulation necrosis, with muscle fibers being visible partially; group B showed a congestion line; and in group C, the fibroid cells did not show degeneration, necrosis, or inflammatory cell infiltration. In the group of samples ablated at 80°C (Figure 2B), the group A exhibited coagulation necrosis with damaged muscle fiber structure, karyoclysis, and reduced nuclear color; group B showed lesser tissue necrosis compared with group A with damaged muscle fiber structure, but with many visible cell nuclei. However, neither muscle cell necrosis nor inflammatory cells infiltration were observed in group C. Additionally, a patent blood vessel was observed in the center of lesion, which showed the presence of red blood cells. In the group of samples ablated with 100°C (Figure 2C), group A exhibited coagulative necrosis, damaged muscle fiber structure, and karyorrhexis; group B demonstrated partial regional necrosis, which was lesser compared with group A, and many cell nuclei were observed. In group C, no obvious fibroid cells degeneration, necrosis or inflammatory cells infiltration were observed. Additionally, several small patent blood vessels with red blood cells were present.

### Tissue Morphological Changes of Uterine Fibroids Treated With In Vivo Radiofrequency Ablation With 100°C

The ablated fibroids were oval in shape, with a sharp demarcation from the peripheral tissue. The center of the ablated lesion was toast tan in color and dry, with no obvious carbonization. The HE stained tissue samples were observed under light microscope (Figure 2D). Group A showed coagluative necrosis, electric shock like fracture, damaged muscle cell structure, and karyorrhexis. In group B, focal coagulative necrosis was observed, the extent of which was lesser compared with group A. The cell spaces were larger in group B compared with group A. In group C, no obvious leiomyoma cell degeneration, necrosis or inflammatory cells infiltration was observed.

### The Effect of Radiofrequency Ablation on ER/PR Expression of In Vitro Uterine Fibroid

**FIGURE 3:**
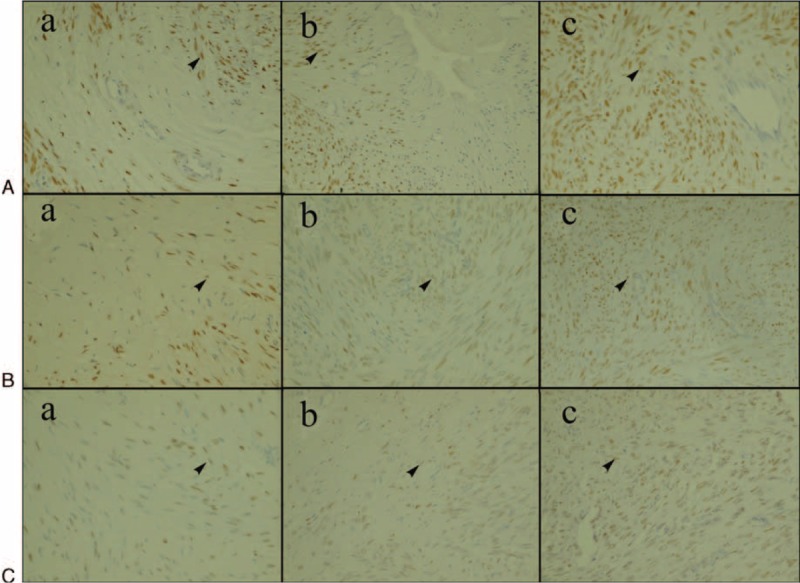
ER expression of fibroid when ablated in vitro at different temperatures (shown as the arrow, ×400). (A) Fibroid ablated at 60°C. (B) Fibroid ablated at 80°C. (C) Fibroid ablated at 100°C. (a) Group A. (b) Group B. (c) Group C. At temperatures 60, 80, and 100°C, the ER optical density was significantly different in the 3 different treatment groups: in group A, it was lesser than in group B, and in group B, it was lesser than in group C. In group A and group B, there were no significant difference in ER optical density between 60 and 80°C treatment groups (*P* > 0.05). However, optical density of ER was higher in 60°C treatment group and 80°C treatment group compared with 100°C treatment group (*P* < 0.05). In group C, there were no significant differences in ER optical density among the 3 temperature groups (*P* > 0.05). ER: estrogen receptor.

The average optical density of ER in the control group and radiofrequency ablation group treated with different temperature is shown in Table 1. At different temperatures, the average optical density in group A, B, and C was lower than that in the control group (*P* < 0.05). At temperatures of 60, 80, 100°C, the ER optical density was significantly different in the three different treatment groups. Additionally, it was lesser in group A than in group B, and in group B than in group C. In group A and group B, there was no significant difference in ER optical density between 60 and 80°C treatment groups (*P* > 0.05). Meanwhile, the optical density of ER was higher in the 60 and 80°C treatment groups compared with that of the 100°C treatment group (*P* < 0.05). In group C, there were no significant difference in ER optical density among 60, 80, and 100°C treatment groups (*P* > 0.05). In conclusion, at the same temperature, the average optical density increased with increasing distance from the electrode. In the same treatment group, the optical density decreased gradually with the increasing treatment temperature. The effect of temperature on ER is shown in Figure 3.

**TABLE 1:**
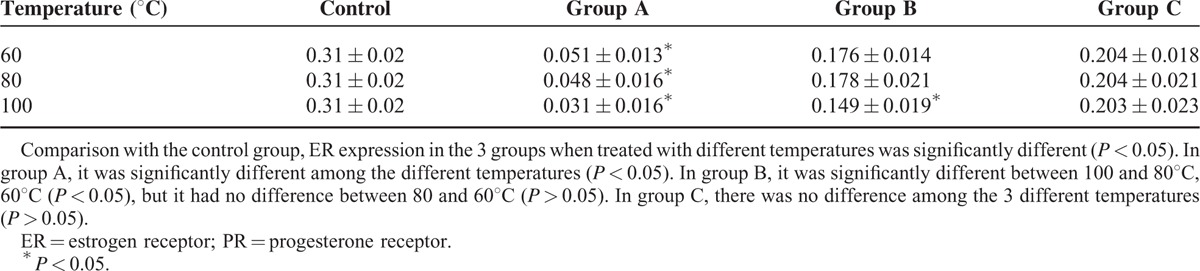
Comparison of ER Average Optical Density of Fibroid When Ablated In Vitro at Different Temperatures

**FIGURE 4:**
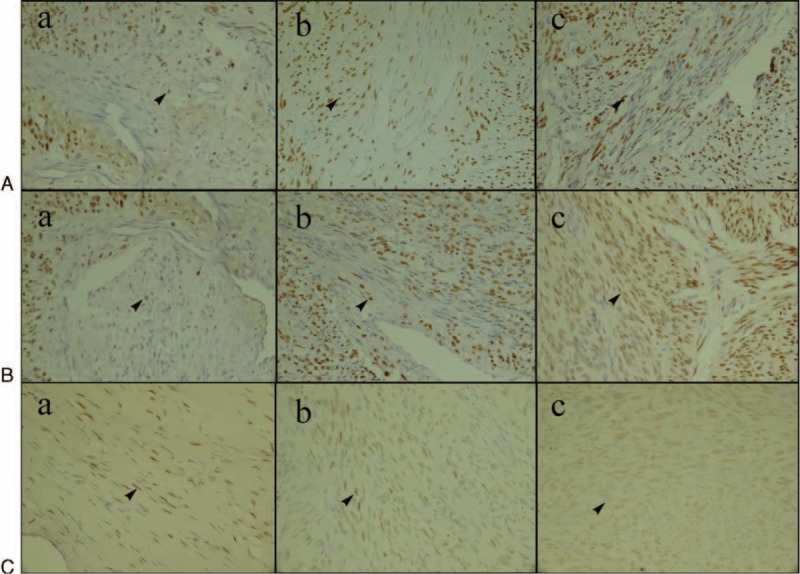
PR expression of fibroid when ablated in vitro at different temperatures (shown as arrow, ×400). (A) Fibroid ablated at a temperature of 60°C. (B) Fibroid ablated at a temperature of 80°C. (C) Fibroid ablated at a temperature of 100°C. (a) Group A. (b) Group B. (c) Group C. In group A, the PR expression decreased with increasing temperature, which was significantly different among the 3 treatment groups ablated at different temperatures (*P* < 0.05). In group B, the PR optical density of the 60°C treatment group was higher than that of 80°C treatment group, and the PR optical density of 80°C treatment group was higher than that of 100°C treatment group. In group C, the PR optical density of 60°C treatment group was higher than that of 80 and 100°C treatment groups. However, the PR optical density between 80 and 100°C treatment groups had no significant difference (*P* > 0.05). PR: progesterone receptor.

The average optical density of PR of the control group and the treatment group ablated with different temperature is shown in Table 2. The PR expression of the 3 treatment groups ablated with different temperature was lower than the control group (*P* < 0.05). In group A, the PR expression decreased with an increasing temperature (*P* < 0.05). In group B, the PR optical density decreased with increasing temperatures. In group C, the PR optical density of 80 and 100°C treatment groups were similar but both were lower than that in 60°C group (*P* > 0.05). In conclusion, the PR optical density of the three treatment groups ablated at different temperatures gradually increased with increasing distance from the electrode. The PR optical density of group A and group B decreased with increasing temperatures (*P* < 0.05). The effect of temperature on PR is shown in Figure 4.

**TABLE 2:**
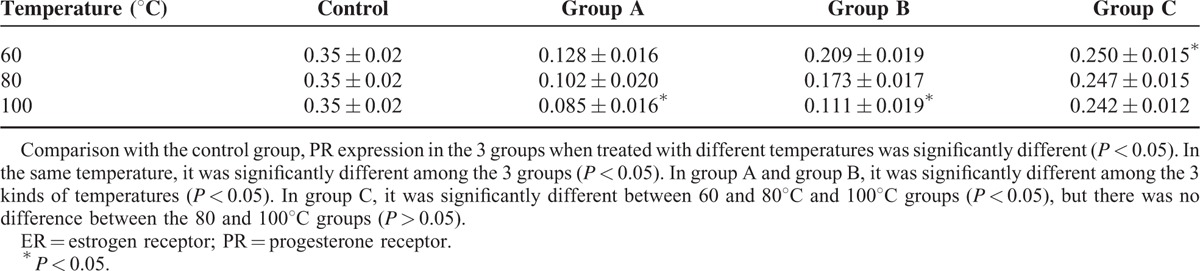
Comparison of PR Average Optical Density of Fibroid When Ablated In Vitro at Different Temperatures

### The Effect of Radiofrequency Ablation on ER/PR Expression of In Vivo Uterine Fibroid

**FIGURE 5:**
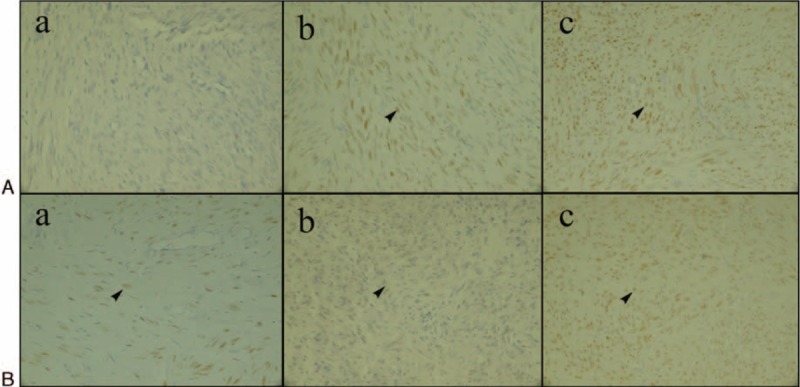
ER/PR expression of fibroid when ablated in vivo at a temperature of 100°C (shown as arrow, ×400). (A) ER expression of fibroid ablated in vivo at a temperature of 100°C. (B) PR expression of fibroid ablated in vivo at a temperature of 100°C. (a) Group A. (b) Group B. (c) Group C. ER/PR optical density in the group A was lesser than that of group B, and the ER/PR optical density in group B was lesser than that of group C, which increased with increasing distance from the electrode.

The ER/PR optical density of the control group and the treatment group ablated with 100°C is shown in Table 3. The PR/ER optical density of the 3 treatment group was lower than that of the control group (*P* < 0.05). Additionally, the ER/PR optical density in the group A was lesser than that of group B, and the ER/PR optical density in the group B was lesser than that of group C, which was increased with the increasing distance from the electrode. These results were accord with in vitro expression. The ER/PR expression is shown in Figure 5.

**TABLE 3:**
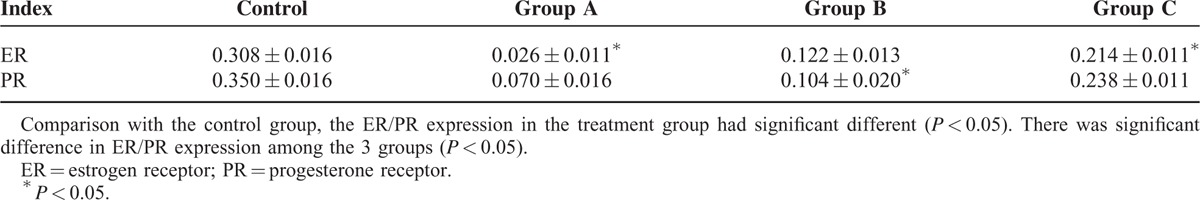
Comparison of ER/PR Average Optical Density of Uterine Fibroid When Ablated In Vivo at a Temperature of 100°C

## DISCUSSION

Radiofrequency ablation makes use of high frequency alternating current, by radiofrequency knife (treatment electrode) piercing into the target tissue, inducing coagulation necrosis and ablation.^13^ Radiofrequency ablation is based on the principle that current density is inversely proportional to the contact area. Here, the positive and negative ions around the small treatment electrode produce high speed vibration affected by the current, which convert mechanical energy into heat energy. Simultaneously, the conduction current converts electrical energy into heat energy by ohm dielectric loss, which increases the local tissue temperature to 80 to 100°C, resulting in coagulative necrosis and in situ inactivation of the target tissue,^14^ with minimal damage to the surrounding tissues.^15^

Radiofrequency ablation treatment of uterine fibroids has the advantage of inducing significant and meaningful reductions in size and consequently symptoms with minimal possible trauma.^16^ The challenge for the radiofrequency ablation is to maximize the coagulative necrosis of the fibroids with minimal or no damage to the surrounding healthy tissue which would eventually reduce the adverse effects.^17^

In our research, the in vitro experiment showed that the treatment effects at 60, 80, and 100°C was different. In this study we could quantify the damage caused by different strengths of current as well as the effect of distance from the electrode. Radiofrequency ablation at a temperature of 100°C had the most severe damage on ER/PR expression in the center and the edge of the ablated lesion. However, the expression at 1 cm from the edge of the ablated tissue had no significant effect on ER expression compared with the control. Ablation at 100°C had more effect on PR expression compared with ablation at 60°C, but compared with ablation with 80°C, there was no significant difference on PR expression. When treating uterine fibroid, too less energy caused incomplete ablation, while too strong energy caused heat damage to surrounding tissue. According to our results, 100°C was the optimal temperature for treating uterine fibroids, which was consistent with other reports.^18,19^

We used a temperature of 100°C, for a duration of 5 minutes and an output power of 30 W to ablate the in vivo fibroid and the results demonstrated coagulative necrosis with minimal ER/PR expression. The edge of the ablated lesion showed focal necrosis, and a significant decrease in the ER/PR expression compared with the control group. At 1 cm distance from the edge, there was no necrosis and the ER/PR expression was similar with in vitro experiment results. Some studies have reported that ablation at 100°C lasting for 35 to 45 seconds could effectively alleviate patient's suffering.^20^ Other studies observed that ablation at 50 Hz with a transmitting power of 300 W and temperatures ranging from 80 to 85°C could significantly improve patient's symptoms.^21^ There are reports of using 400 kHz with a maximum power of 120 W at temperatures ranging from 40 to 99°C for effective ablation of fibroids.^22^

In conclusion, we have shown with in vitro and in vivo experiments that radiofrequency ablation at temperature of 100°C, for a duration of 5 minutes and the output power of 30 W is adequate for ablating uterine fibroids. This has established the safety of the surrounding normal tissues. We have established the ablation with demonstration of coagulative necrosis and diminished ER/PR expression which was directly proportional to the distance from the electrode as well as the temperature. Further research is needed to establish the clinical safety and preservation of reproduction functions after RFA.
